# A suspected case of “itai-itai disease” in a cadmium-polluted area in Akita prefecture, Japan

**DOI:** 10.1265/ehpm.24-00063

**Published:** 2024-08-07

**Authors:** Toru Sasaki, Hyogo Horiguchi, Takehisa Matsukawa, Momoko Kobayashi, Yuki Omori, Etsuko Oguma, Atsushi Komatsuda

**Affiliations:** 1 Fukunaga Clinic, Akita 018-5334, Japan; 2 Akita Rosai Hospital, Japan Organization of Occupational Health and Safe, Akita 018-5604, Japan; 3 Department of Hygiene, Kitasato University School of Medicine, Kanagawa 252-0374, Japan; 4 Department of Epidemiology and Environmental Health, Juntendo University Faculty of Medicine, Tokyo 113-8421, Japan; 5 Department of Hematology, Nephrology, and Rheumatology, Akita University Graduate School of Medicine, Akita 010-8543, Japan; 6 Ogachi Central Hospital, Akita 012-0055, Japan

**Keywords:** Itai-itai disease, Cadmium, Akita prefecture

## Abstract

**Background:**

Itai-itai disease is the most severe case of chronic cadmium (Cd) toxicity, which was endemic in Cd-polluted areas in the Jinzu River basin in Toyama prefecture, Japan. Akita prefecture also has Cd-polluted areas, but there have been no cases of “itai-itai disease”.

**Case presentation:**

An elderly female farmer with Cd nephropathy residing in a Cd-polluted area in the northern part of the Akita prefecture was identified through hospital-based screening at Akita Rosai Hospital in Odate city. She had chronic renal failure with a high Cd exposure level and advanced renal tubular dysfunction. The shortening of height, bone deformities and fractures, abnormal bone metabolism suggesting osteomalacia, and renal anemia were also noted. Therefore, “itai-itai disease”, similar to cases in the Jinzu River basin, was suspected.

**Conclusion:**

This is the first case of “itai-itai disease” in a Cd-polluted area in Akita prefecture.

## Introduction

“Itai-itai disease” is the most severe case of chronic cadmium (Cd) toxicity and was endemic during the 20th century in the Jinzu River basin in Toyama prefecture, Japan, where rice paddy fields were heavily polluted by Cd derived from the Kamioka mine upstream in adjacent Gifu prefecture [1, 2]. Patients with itai-itai disease, most of whom were female farmers, were highly exposed to Cd through the consumption of self-harvested rice and river water. They developed osteomalacia, severe pain throughout the body (“itai” means “ouch”), and bone deformities and fractures. Bone injuries occur secondary to Cd-induced proximal renal tubular dysfunction (Cd nephropathy), which is characterized by a decrease in the renal reabsorption of, for example, water, glucose, low-molecular weight proteins, phosphorus, calcium, and bicarbonate ions. Hypophosphatemia due to the urinary loss of phosphorus is the main cause of osteomalacia. Therefore, itai-itai disease is diagnosed using 3 criteria: environmental exposure to Cd, renal tubular dysfunction, and osteomalacia [3]. By 2022, 201 patients with itai-itai disease were officially recognized in the Jinzu River basin by the governor of Toyama prefecture [4].

Several areas outside the Jinzu River basin were polluted by Cd through the activity of mines or smelters in Japan in the 20th century. Representative examples are the Kakehashi River basin in Ishikawa prefecture [5], Tsushima island in Nagasaki prefecture [6], the Ichi River basin in Hyogo prefecture [7], and Kosaka town in Akita prefecture [8]. While patients with Cd nephropathy were reported in these areas, those with osteomalacia, who could be considered to have had “itai-itai disease”, were not always identified. Osteomalacia was histologically confirmed in 9 autopsy cases in Tsushima island [9], and X-ray photographs of bones in 5 patients showed Looser zones (translucent fracture lines with adjacent scleroses on both sides), which are specific to osteomalacia [10], in the Ichi River basin [11]. However, any of cases of Cd nephropathy in the Kakehashi River basin or Kosaka town did not present signs of osteomalacia.

Kosaka town was investigated in the 1970s as one of the Cd-polluted areas in Akita prefecture; it had the widest Cd-polluted area in Japan formally designated by the Anti-Farm Soil Pollution Law, with almost the same width as that in Toyama prefecture [12]. In contrast to the Jinzu River basin, Cd-polluted areas in Akita prefecture were scattered and, thus, were divided into northern, central and southern parts, with Kosaka town belonging to the northern part. Health examinations were performed for inhabitants in the northern part, consisting of Odate city, Kazuno city, and Kosaka town, in 2001–2004, and the findings obtained confirmed exposure to various levels of Cd and renal tubular dysfunction in elderly females exposed to high levels [13].

In addition to health examinations for local inhabitants, hospital-based screening to detect Cd nephropathy has been performed at medical institutes in northern Cd-polluted areas in Akita prefecture since 2010. Outpatients who are elderly farmers with reduced renal function (an elevated serum creatinine concentration) are selected for screening and their urinary β_2_-migroglobulin (β2MG) (a low-molecular-weight protein as an indicator for renal tubular dysfunction), blood Cd, and urinary Cd concentrations are measured [14]. When these levels are elevated, Cd nephropathy is suspected and they are followed up by detailed examinations that focus on secondary osteomalacia. This screening at medical institutes effectively detects patients with Cd nephropathy.

We herein present a case of Cd nephropathy detected by screening with clinical features that were very close to those of itai-itai disease, and discuss whether this is an actual case of “itai-itai disease”.

## Case presentation

The patient was a female farmer who had lived in a farming area in Odate city from birth. She had no history of smoking, hypertension, or diabetes. The hamlet of her home village is located near the Osarizawa mine in Kazuno city and paddy fields in the area were polluted by Cd in the river coming from the mine [12]. Therefore, she was assumed to have consumed locally harvested rice and vegetables contaminated with Cd.

In May 1995, she was hospitalized in the Orthopedic Department at Akita Rosai Hospital in Odate city and underwent right high tibial osteotomy based on the diagnosis of spontaneous osteonecrosis of the right knee, leaving tibial pseudoarthrosis. At that time, she was 66 years old and her height and weight were 147.5 cm and 46.0 kg, respectively. Clinical examinations showed a serum creatinine concentration of 0.78 mg/dL, blood urea nitrogen of 17.7 mg/dL, and a hemoglobin concentration of 12.9 g/dL. The results of qualitative urinalyses were protein − and occult blood +/−. In July 2000, aged 71 years, she consulted the Orthopedic Department at Akita Rosai Hospital with chronic lower back pain. An X-ray examination showed multiple spinal compression fractures, based upon which she was diagnosed with osteoporosis. She started outpatient visits to the hospital, receiving activated vitamin D3, calcium L-aspartate, and elcatonin. During treatment, the patient underwent surgical repair for a femoral hernia in September 2005 aged 76 years.

In September 2007, aged 78 years, she was hospitalized in the Internal Department at Akita Rosai Hospital with an acute respiratory infection. Her height and weight at this time were 135.0 cm and 38.0 kg, respectively, indicating the severe shortening of height in addition to kyphosis. Clinical examinations showed a blood pressure of 108/67 mmHg, pulse rate of 92/min, SpO_2_ of 97% (room temperature), serum creatinine concentration of 2.02 mg/dL, blood urea nitrogen of 20.8 mg/dL, 24-h creatinine clearance of 19.9 mL/min, hemoglobin concentration of 7.6 g/dL, serum erythropoietin concentration of 17.7 mIU/mL, and serum ferritin concentration of 342 ng/mL. The results of qualitative urinalyses were urine-specific gravity of 1.015, pH of 6.0, protein 2+, occult blood 1+, and sugar 2+. Based on these results, the patient was diagnosed with chronic renal failure and renal anemia; therefore, treatment with an erythropoietin preparation was initiated. In addition, treatment for osteoporosis was changed to alendronate sodium hydrate at the Orthopedic Department because of hypercalcemia, which may have been caused by the previous treatment. Treatment continued until 2015. One of the follow-up examinations in November 2008 at the age of 79 years when she was hospitalized with a pubic fracture after falling down showed a height of 134.0 cm, weight of 35.0 kg, serum creatinine concentration of 2.07 mg/dL, blood urea nitrogen of 45.7 mg/dL, and hemoglobin concentration of 9.8 g/dL. The results of qualitative urinalyses were protein 1+ and occult blood +/−.

Hospital-based screening to detect Cd nephropathy started at the Internal Department of Akita Rosai Hospital in 2010 [14], and the patient was listed as a candidate in August based on her history and elevated serum creatinine concentration of 2.05 mg/dL at the age of 81 years. The results of examinations showed a urinary β2MG concentration of 12,600 µg/g cr., blood Cd concentration of 11.3 µg/L, and urinary Cd concentration of 5.93 µg/g cr. Cd concentrations were measured using inductively coupled plasma-mass spectrometry (ICP-MS) Agilent 8800 (Agilent Technologies, Tokyo, Japan) as described in detail in a previous study [14]. Since we set the criteria for Cd nephropathy as a urinary β2MG concentration ≥10,000 µg/g cr. and blood Cd concentration ≥6 µg/L or urinary Cd ≥10 µg/g cr. as stated in the reference 14, Cd nephropathy was suspected and, thus, we continued to follow up the patient. She did not have edema at that time, and additional qualitative urinalyses showed protein 1+, occult blood 1+, and sugar −.

In October 2015, aged 86 years, the acute worsening of renal failure and decreased physical strength from unknown reasons were noted, and the patient was hospitalized in the Internal Department at Akita Rosai Hospital. At admission, she presented with consciousness disorder and generalized edema, and her height and weight were 130.7 cm and 41.5 kg, respectively. Clinical examinations showed a serum creatinine concentration of 8.18 mg/dL and blood urea nitrogen of 105.5 mg/dL (they had remained at 3.5–4.0 and 40–50 mg/dL, respectively, for approximately 1 year). At discharge from the hospital after treatment for approximately 5 months, her serum creatinine concentration and blood urea nitrogen decreased to 4.07 and 39.4 mg/dL, respectively. She continued regular visits to the hospital. In February 2017, aged 88 years, clinical examinations showed a serum creatinine concentration of 5.32 mg/dL and blood urea nitrogen of 66.4 mg/dL, suggesting the gradual worsening of renal function and an elevated serum creatinine concentration >5.0 mg/dL.

**Fig. 1:**
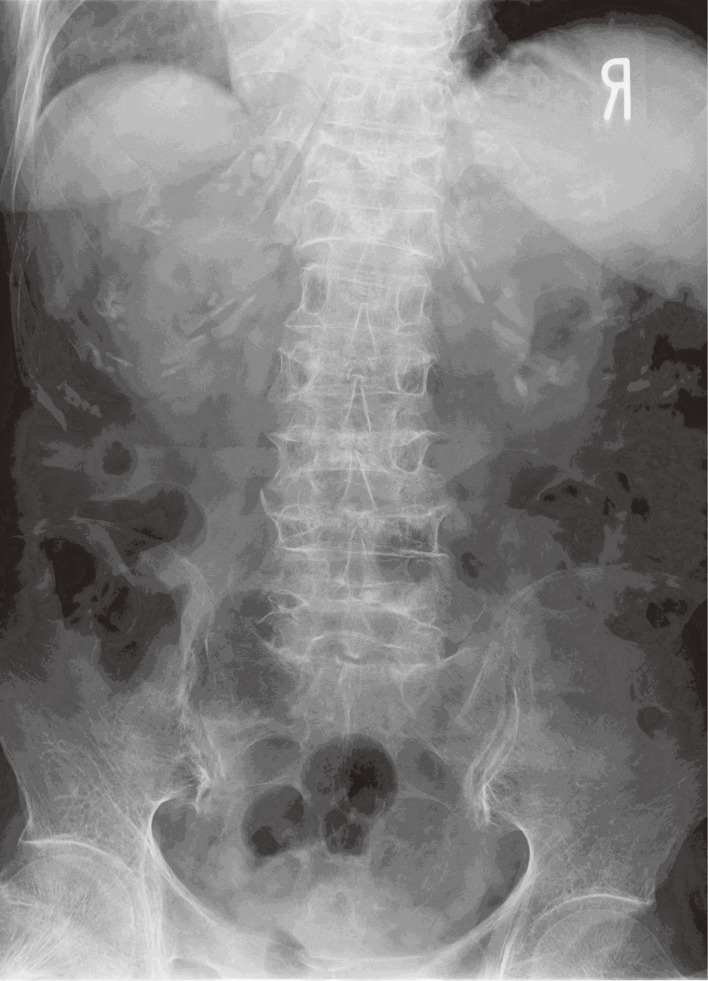
An X-ray photograph of thoracic and lumbar vertebrae from the front.

**Fig. 2:**
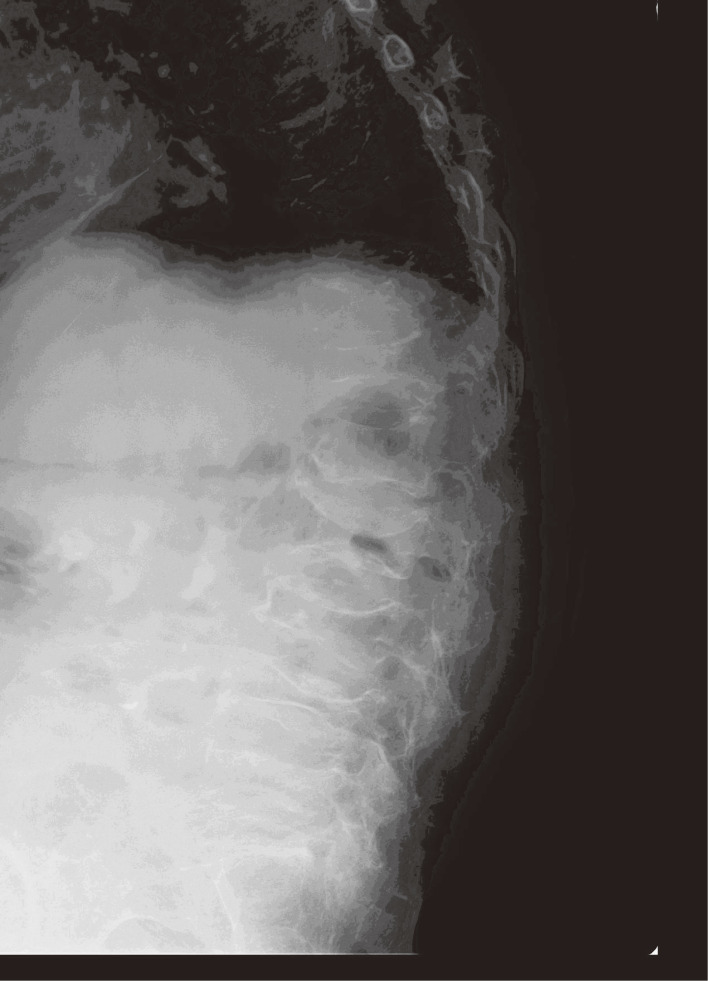
An X-ray photograph of thoracic and lumbar vertebrae from the side.

Due to the gradual worsening of renal function, examinations for screening were performed in November 2017 when the patient was 88 years, and showed a serum creatinine concentration of 5.15 mg/dL, urinary β2MG concentration of 48,700 µg/g cr., blood Cd concentration of 10.3 µg/L, and urinary Cd concentration of 3.74 µg/g cr. Since her condition was deteriorating with the progression of renal tubular dysfunction and height shortening, “itai-itai disease” was suspected and, thus, detailed examinations for renal dysfunction, osteomalacia, and anemia were performed at the Internal Department of Akita Rosai Hospital in October 2018 at the age of 89 years (Table 1). The results obtained showed a total serum protein concentration of 6.3 g/dL, serum albumin concentration of 3.6 g/dL, HbA1c concentration of 4.7%, erythrocyte count of 248 × 10^4^/µL, hemoglobin concentration of 8.2 g/dL, hematocrit of 25.7%, mean corpuscular volume (MCV) of 103.6 fL, mean corpuscular hemoglobin (MCH) of 33.1 pg, mean corpuscular hemoglobin concentration (MCHC) of 31.9%, reticulocyte count rate of 22.3‰, serum ferritin concentration of 62 ng/mL, serum transferrin concentration of 174 mg/dL, serum creatinine concentration of 5.91 mg/dL, blood urea nitrogen of 56.6 mg/dL, serum cystatin C concentration of 4.3 mg/L, estimated glomerular filtration rate (GFR) from serum creatinine (eGFRcr) of 5.7 mL/min/1.73 m^2^, serum phosphorus concentration of 3.2 mg/dL, urinary phosphorus concentration of 16.3 mg/dL, urinary creatinine concentration of 45.95 mg/dL, urinary α_1_-microglobulin (α1MG) concentration of 231 mg/g cr., urinary β2MG concentration of 178,000 µg/g cr., retinol-binding protein (RBP) concentration of 106.6 mg/g cr., tubular reabsorption of phosphate (TRP) of 34.5%, the ratio of the tubular maximum reabsorption of phosphate (TmP) to GFR (TmP/GFR) of 1.10 mg/dL, serum sodium concentration of 143.3 mEq/L, serum potassium concentration of 4.3 mEq/L, serum chlorine (Cl) concentration of 117.7 mEq/L, serum calcium (Ca) concentration of 8.2 mg/dL (since the serum albumin concentration was low, this value was corrected to 8.1 mg/dL, according to the Kidney Disease Outcomes Quality Initiative–2 formula [15]), serum alkaline phosphatase concentration of 499 IU/L, bone-specific alkaline phosphatase concentration of 48.4 µg/L, whole-parathyroid hormone (PTH) concentration of 204 pg/mL, and urinary *N*-telopeptide crosslinked collagen type 1 (NTx) concentration of 90.1 nmol/mmol cr. The results of qualitative urinalyses were pH of 7.0, urine-specific gravity of 1.011, protein 1+, and glucose 3+. X-ray photographs of bones showed general atrophy and thinning of the bone cortex, compression fractures and flattening of the cervical, thoracic, and lumbar vertebrae (Figs. 1 & 2), tibial pseudoarthrosis, and crooked femurs, but no Looser zone.

**Table 1:** Results of clinical examinations on the patient at 89.

**Examination items**	**Results**	**Reference ** **value**
Total serum protein (g/dL)	6.3	6.6–8.1
Serum albumin (g/dL)	3.6	4.1–5.1
HbA1c (%)	4.7	4.9–6.0
Erythrocyte count (×10^4^/µL)	248	386–492
Hemoglobin (g/dL)	8.2	11.6–14.8
Hematocrit (%)	25.7	35.1–44.4
Mean corpuscular volume (fL)	103.6	83.6–98.2
Mean corpuscular hemoglobin (pg)	33.1	27.5–33.2
Mean corpuscular hemoglobin concentration (%)	31.9	31.7–35.3
Reticulocyte count rate (‰)	22.3	5–17
Serum ferritin (ng/mL)	62	12–60
Serum transferrin (mg/dL)	174	190–320
Serum creatinine (mg/dL)	5.91	0.46–0.79
Blood urea nitrogen (mg/dL)	56.6	8–20
Serum cystatin C (mg/L)	4.3	0.51–0.82
eGFRcr (mL/min/1.73 m^2^)	5.7	≥60
Serum phosphorus (mg/dL)	3.2	2.7–4.6
Urinary phosphorus (mg/dL)	16.3	
Urinary creatinine concentration (mg/dL)	45.95	
Urinary α_1_-microglobulin (mg/g cr.)	231	
Urinary β_2_-microglobulin concentration (µg/g cr.)	178,000	
Retinol-binding protein (mg/g cr.)	106.6	
Tubular reabsorption of phosphate (%)	34.5	≥80
TmP/GFR (mg/dL)*	1.10	≥2.5
Serum sodium (mEq/L)	143.3	138–145
Serum potassium (mEq/L)	4.3	3.6–4.8
Serum chlorine (mEq/L)	117.7	101–108
Serum calcium (mg/dL)	8.2	8.8–10.1
Serum alkaline phosphatase (IU/L)	499	106–322
Bone-specific alkaline phosphatase (µg/L)	48.4	3.8–22.6
Whole-parathyroid hormone (pg/mL)	204	8.3–38.7
NTx (nmol/mmol cr.)	90.1	>55
Urinary pH	7.0	
Urine-specific gravity	1.011	
Urinary protein	1+	
Urinary glucose	3+	

*: the ratio of the tubular maximum reabsorption of phosphate (TmP) to GFR

Her renal condition continued to deteriorate and she was transferred and admitted to Odate Municipal General Hospital for more intensive care for renal failure in November 2018 aged 89 years. However, she presented with generalized edema, pulmonary edema, a serum creatinine concentration of 7.08 mg/dL, and eGFR of 4.6 mL/min/1.73 m^2^. The continuous administration of diuretics was ineffective and the patient died of renal failure 10 days after admission.

## Discussion

Our patient developed osteoporosis and bone deformities and fractures from her 60s. Furthermore, her height was shortening and the worsening of renal function and renal anemia were detected for the first time at the age of 78 years. Renal tubular dysfunction was noted and Cd nephropathy was suspected by hospital-based screening at the age of 81 years. Renal glomerular and tubular functions both gradually worsened, along with impaired bone metabolism and bone injuries, and the patient died at the age of 89 years (Table 2).

**Table 2:** Progression of the patient’s disease state.

**Age**	**66**	**78**	**81**	**86**	**88**	**89**
Height (cm)	147.5	135.0		130.7		
Weight (kg)	46.0	38.0		41.5		
Serum creatinine (mg/dL)	0.78	2.02	2.05	8.18 (4.07 after treatment)	5.15	5.91 (7.08 just before death)
Creatinine clearance (mL/min)/eGFRcr (mL/min/1.73 m^2^)		19.9				5.7 (4.6 just before death)
Hemoglobin (g/dL)	12.9	7.6				8.2
Urinary β_2_-nicroglobulin (µg/g cr.)			12,600		48,700	178,000
Blood cadmium (µg/L)			11.3		10.3	
Urinary cadmium (µg/g cr.)			5.93		3.74	
Notes		Erythropoietin preparation treatment initiated	Examined as hospital-based screening	Hospitalized and treated	Examined as hospital-based screening	

Her blood Cd concentration was very high, while her urinary Cd concentration was low, both of which were repeatedly measured at the ages of 81 and 88 years. Patients with highly advanced renal tubular dysfunction due to Cd exposure, such as itai-itai disease, generally have low Cd concentrations in the kidney unexpectedly due to the loss of damaged renal tubular cells, and, thus, the low renal excretion of Cd. In these cases, the blood concentration of Cd is used as an adequate indicator of the accumulation of Cd in the body. A previous study reported that approximately 10% of rice produced in the Cd-polluted area in Odate city was over the safe standard for Cd concentrations until 2002, before measures for Cd pollution, such as the flooding of paddy fields, had been taken [13]. Therefore, the patient accumulated a high concentration of Cd in her body, which may have been attributed to the lifelong and continuous consumption of self-harvested rice with a high Cd concentration.

A decrease in renal glomerular function was initially detected when the patient was 78 years, and the initial measurement of the urinary concentration of β2MG at the age of 81 years by hospital-based screening revealed that it was elevated (12,600 µg/g cr.), indicating the deterioration of renal tubular function. However, renal tubular dysfunction may already have occurred at the age of 78 years because the urinalysis of sugar was 2+ at that time, suggesting renal glycosuria (the renal excretion of glucose without diabetes due to impaired reabsorption at the renal tubules). Renal tubular function continued to deteriorate, with a urinary β2MG concentration of 48,700 µg/g cr. being measured at the age of 88 years. In the last stage of life, aged 89 years, the urinary concentration of β2MG rapidly increased to 178,000 µg/g cr., in addition to elevations in the urinary concentration of α1MG and RBP and reductions in TRP (34.5%, reference of ≥80%) and TmP/GFR (1.10 mg/dL, reference of ≥2.5 mg/dL), indicating significantly advanced renal tubular dysfunction. Furthermore, low urine-specific gravity of 1.011, indicating an increase in the volume of urine, as well as renal glycosuria supported the decrease in tubular reabsorption. In addition, the high serum Cl concentration (117.7 mEq/L, reference of 101–108 mEq/L) and high urinary pH of 7.0 suggested metabolic acidosis due to the urinary loss of bicarbonate ions. In contrast, hypophosphatemia, which is a significant clinical feature of renal tubular dysfunction, was not observed (3.2 mg/dL, reference of 2.7–4.6 mg/dL). However, based on the worsening of renal glomerular function (eGFR 5.7 mL/min/1.73 m^2^, reference of ≥60 mL/min/1.73 m^2^), which may elevate the serum concentration of phosphorus, the value may be assumed to indicate “covert hypophosphatemia”. The patient had no history of the prolonged administration of analgesics, multiple myeloma, and treatment for chronic hepatitis, which may induce renal tubular injury. Similar clinical conditions occur in itai-itai disease, the last stage of Cd-induced renal tubular dysfunction [16]. These results indicate that the patient had Cd-induced renal tubular dysfunction, namely, Cd nephropathy.

In addition to osteoporosis, the patient presented with abnormal bone metabolism characteristic of osteomalacia: covert hypophosphatemia, a low serum concentration of Ca (8.1 mg/dL, reference of 8.8–10.1 mg/dL), elevated serum bone-specific alkaline phosphatase (48.4 µg/L, reference of 3.8–22.6 IU/L), elevated serum whole-PTH (204 pg/mL, reference of 8.3–38.7 pg/mL), and elevated urinary NTx (90.1 nmol/mmol cr., reference of >55 nmol/mmol cr.), which is an osteoclastic marker. Hypophosphatemia and elevated serum alkaline phosphatase concentrations are observed in typical cases of Cd nephropathy; however, serum Ca and PTH concentrations are generally within normal ranges [17, 18]. In the present case, secondary hyperparathyroidism due to severe renal glomerular dysfunction was assumed to have increased the serum concentration of phosphorus and reduced that of Ca. These changes in bone metabolism indicated that the patient developed osteomalacia due to renal tubular dysfunction, which was supported by the lack of a history of gastrointestinal surgery, the administration of acid suppressants, and malnutrition, which may induce osteomalacia. The patient’s physical appearance, such as the shortening of height and kyphosis, and bone atrophy and various bone injuries detected by X-ray photographs, further suggested osteomalacia.

In Toyama prefecture, itai-itai disease is diagnosed according to the following criteria laid down by the Ministry of the Environment of Japan in 1972: “(1) a history of environmental exposure to Cd derived from living in a heavily Cd-polluted area (in the Jinzu River basin, though not specified), (2) a non-congenital clinical status of (3) and (4) that occurred after adolescence, mainly after menopause, (3) the recognition of renal tubular injury, (4) the recognition of clinical findings of osteomalacia accompanied by osteoporosis in an X-ray examination, biopsy, or autopsy of bones, such as atrophy, Looser zones, deformity of bones, etc. Even in the case that osteomalacia is not confirmed just by these bone findings, the results of clinical examinations, including serum phosphorus, Ca, and alkaline phosphatase concentrations, that are consistent with osteomalacia are considered as clinical findings of osteomalacia, in addition to bone findings to suggest osteomalacia.” (the Environment Agency in Japan, 1972). The patient had a history of living in the Cd-polluted area in Akita, renal tubular dysfunction, abnormal bone metabolism, and bone atrophy and deformity. Although no Looser zone was not recognized, the patient obviously presented with osteomalacia evidenced by the clinical examinations in addition to the suggestive bone findings. Therefore, since this patient fulfilled all of these criteria, it is not unreasonable to suspect that she had “itai-itai disease” despite the case been detected in Akita, not in the Jinzu River basin.

On the other hand, the patient had advanced renal anemia, as evidenced by the normal serum concentration of erythropoietin (17.7 mIU/mL, reference of 9.1–32.8 mIU/mL) despite a severely reduced concentration of hemoglobin (7.6 g/dL, reference of 11.6–14.8 g/dL) when anemia was initially detected at the age of 78 years. Although the patient was being treated with an erythropoietin preparation, a decreased erythrocyte count (248 × 10^4^/µL, reference of 386–492 × 10^4^/µL), hemoglobin concentration (8.2 g/dL, reference of 11.6–14.8 g/dL), and hematocrit (25.7%, reference of 35.1–44.4%) were still observed in the very last stage of life at the age of 89 years. Anemia was macrocytic and normochromic, as evidenced by the elevated concentration of MCV (103.6 fL, reference of 83.6–98.2 fL), and the normal concentrations of MCH (33.1 pg, reference of 27.5–33.2 pg) and MCHC (31.9%, reference of 31.7–35.3%). In addition, her serum ferritin concentration (62 ng/mL, reference of 12–60 ng/mL) was not low, it was slightly high, while her serum transferrin concentration (174 mg/dL, reference of 190–320 mg/dL) was low, not elevated, indicating that anemia was not derived from an iron deficiency. Furthermore, the reticulocyte count rate (22.3‰, reference of 5–17‰) was not decreased, it was slightly elevated, indicating no involvement of bone marrow dysfunction in anemia. Previous studies showed that anemia in itai-itai disease was attributed to progressive renal damage, which resulted in the hypoproduction of erythropoietin [19], providing further support for the patient having “itai-itai disease”. Although the current criteria for itai-itai disease do not include renal anemia, its inclusion has been recommended [20, 21].

Therefore, this is the first case report of “itai-itai disease” from a Cd-polluted area in Akita prefecture.

## Conclusion

An elderly female farmer with Cd nephropathy was detected by hospital-based screening in a Cd-polluted area in Akita prefecture, Japan. The shortening of height, bone deformities and fractures, a high Cd exposure level, advanced renal tubular dysfunction, abnormal bone metabolism that suggested osteomalacia, and renal anemia were noted. Therefore, the patient was suspected to have “itai-itai disease”, which is the first reported case in Akita prefecture.

## Abbreviations

Cd: cadmium
α1MG: α_1_-microgloburin
β2MG: β_2_-microglobulin

## Declarations

### Availability of data and materials

The datasets used during the present study are available from the corresponding author upon reasonable request.

## References

[1] Nordberg GF, Åkesson A, Nogawa K, Nordberg M. Cadmium. In: Nordberg GF, Costa M, editors. Handbook on the Toxicology of Metals. 5th ed. Volume II. Specific Metals. Burlington: Academic Press; 2022. p. 141–96.

[2] Aoshima K, Horiguchi H. Historical lessons on cadmium environmental pollution problems in Japan and current cadmium exposure situation. In: Himeno S, Aoshima K, editors. Cadmium Toxicity. Singapore: Springer Nature Singapore; 2019. p. 12–9.

[3] Aoshima K. Itai-itai disease: Cadmium-induced renal tubular osteomalacia –Current situations and future perspectives–. Jpn J Hyg. 2012;67:455–63 (in Japanese with English abstract). 10.1265/jjh.67.455 23095355

[4] Aoshima K. History of official recognition of patients with itai-itai disease. Jpn J Hyg. 2023;78(supplement):S123 (in Japanese).

[5] Nogawa K, Suwazono Y, Kido T. Long-term follow-up study of residents exposed to cadmium in Kakehashi River basin, Ishikawa, Japan. In: Himeno S, Aoshima K, editors. Cadmium Toxicity. Singapore: Springer Nature Singapore; 2019. p. 51–62.

[6] Iwata K, Saito H, Moriyama M, Nakano A. Association between renal tubular dysfunction and mortality among residents in a cadmium-polluted area, Nagasaki, Japan. Tohoku J Exp Med. 1991;164:93–102. 1750038 10.1620/tjem.164.93

[7] Nogawa K, Ishizaki A, Kobayashi E, Inaoka H, Shibata I. Studies on renal dysfunction of inhabitants in Cd-polluted district. Jpn J Hyg. 1975;30:549–55 (in Japanese with English abstract). 176488

[8] Saito H, Shioji R, Hurukawa Y, Nagai K, Arikawa T. Cadmium-induced proximal tubular dysfunction in a cadmium-polluted area. Contrib Nephrol. 1977;6:1–12. 192520 10.1159/000399746

[9] Takebayashi S, Jimi S, Segawa M, Kiyoshi Y. Cadmium induces osteomalacia mediated by proximal tubular atrophy and disturbances of phosphate reabsorption. A study of 11 autopsies. Pathol Res Pract. 2000;196:653–63. 10997741 10.1016/S0344-0338(00)80010-2

[10] Yamashita H, Kitagawa M. Histomorphometric study of ribs with looser zones in Itai-itai disease. Calcif Tissue Int. 1996;58:170–6. 8852572 10.1007/BF02526883

[11] Nogawa K, Ishizaki A, Fukushima M, Shibata I, Hagino N. Studies on the women with acquired Fanconi syndrome observed in the Ichi river basin polluted by cadmium. Is this Itai-itai disease? Environ Res. 1975;10:280–307. 172326 10.1016/0013-9351(75)90090-0

[12] Horiguchi H. Cadmium exposure and its effects on the health status of rice farmers in Akita prefecture. In: Himeno S, Aoshima K, editors. Cadmium Toxicity. Singapore: Springer Nature Singapore; 2019. p. 75–83.

[13] Horiguchi H, Oguma E, Sasaki S, Okubo H, Murakami K, Miyamoto K, . Age-relevant renal effects of cadmium exposure through consumption of home-harvested rice in female Japanese farmers. Environ Int. 2013;56:1–9. 23542681 10.1016/j.envint.2013.03.001

[14] Sasaki T, Horiguchi H, Arakawa A, Oguma E, Komatsuda A, Sawada K, . Hospital-based screening to detect patients with cadmium nephropathy in cadmium-polluted areas in Japan. Environ Health Prev Med. 2019;24:8. 30684957 10.1186/s12199-019-0762-3 PMC6347770

[15] Wang L, Yoine S, Sokawa S, Yamada S, Kuwahara T. Evaluation of calcium correction formulae in hemodialysis patients. J Jpn Soc Dial Ther. 2018;51:103–7 (in Japanese with English abstract).

[16] Kasuya M, Aoshima K, Katoh T, Teranishi H, Horiguchi H, Kitagawa M, et al. Natural history of ltai-itai disease: A longterm observation on the clinical and laboratory findings in patients with ltai-itai disease. In: Cook ME, Hiscock SA, Morrow H, Volpe RA, editors. Edited Proceedings Seventh International Cadmium Conference New Orleans. London: Cadmium Association, Reedprint Limited; 1992. p. 180–92.

[17] Aoshima K, Iwata K, Kasuya M. Environmental exposure to cadmium and effects on human health part 2. Bone and mineral metabolism in inhabitants of the cadmium-polluted Jinzu River basin in Toyama prefecture. Jpn J Hyg. 1988;43:864–71 (in Japanese with English abstract). 10.1265/jjh.43.864 3249418

[18] Aoshima K, Katoh T, Teranishi H, Horiguchi H, Kasuya M. Abnormalities of calcium, phosphorus, and vitamin D metabolism with proximal renal tubular dysfunction in subjects environmentally exposed to cadmium. Jpn J Hyg. 1988;47:1009–20 (in Japanese with English abstract). 10.1265/jjh.47.1009 8492479

[19] Horiguchi H, Teranishi H, Niiya K, Aoshima K, Katoh T, Sakuragawa N, . Hypoproduction of erythropoietin contributes to anemia in chronic cadmium intoxication: clinical study on Itai-Itai disease in Japan. Arch Toxicol. 1994;68:632–6. 7857202 10.1007/BF03208342

[20] Horiguchi H, Aoshima K, Oguma E, Sasaki S, Miyamoto K, Hosoi Y, . Latest status of cadmium accumulation and its effects on kidneys, bone, and erythropoiesis in inhabitants of the formerly cadmium-polluted Jinzu River Basin in Toyama, Japan, after restoration of rice paddies. Int Arch Occup Environ Health. 2010;83:953–70. 20130905 10.1007/s00420-010-0510-x

[21] Horiguchi H. Anemia induced by cadmium intoxication. Jpn J Hyg. 2007;62:888–904 (in Japanese with English abstract). 10.1265/jjh.62.888 17575787

